# Association between serum 25‐hydroxyvitamin D concentrations and metabolic syndrome in the middle‐aged and elderly Chinese population in Dalian, northeast China: A cross‐sectional study

**DOI:** 10.1111/jdi.13086

**Published:** 2019-06-21

**Authors:** Tesfaye Zerfu Weldegiorgis, Tesfaldet Habtemariam Hidru, Xiao‐lei Yang, Yun‐long Xia, Li Ma, Hui‐Hua Li

**Affiliations:** ^1^ School of Public Health Dalian Medical University Dalian China; ^2^ Department of Cardiology Institute of Cardiovascular Diseases First Affiliated Hospital of Dalian Medical University Dalian China

**Keywords:** 25‐Hydroxyvitamin D, Metabolic syndrome, Vitamin D

## Abstract

**Aims/Introduction:**

To investigate the association between serum 25‐hydroxyvitamin D (25‐[OH]D) concentrations and metabolic syndrome (MetS) in the middle‐aged and elderly Chinese population.

**Methods:**

The present study included 2,764 participants (aged >50 years). The joint interim statement was used for the standard definition of MetS. Serum 25‐(OH)D concentrations were measured by electrochemiluminescence immunoassay. The study participants were categorized into quartiles based on serum 25‐(OH)D concentrations, and the quartiles were calculated for the differences using anova and the χ^2^‐test for continuous and categorical data, respectively. A logistic regression analysis model was applied to estimate the odds ratios and 95% confidence intervals (95% CI) for each quartile of serum 25‐(OH)D concentrations compared with the highest quartile.

**Results:**

Serum 25‐(OH)D levels were markedly lower in men in the MetS group than in those without MetS. We observed a negative correlation between the higher quartiles of serum 25‐(OH)D levels and the presence of MetS among men. The correlation between serum 25‐(OH)D levels and the prevalence of MetS persisted even after adjusting for potential confounders, including age, cigarette smoking status, alcohol consumption, physical activity, low‐density lipoprotein, creatinine and total serum cholesterol. Adjusted odds ratios of MetS in the second through fourth compared with the lowest quartile for serum 25‐(OH)D levels were 0.93 (95% CI 0.54–1.59), 0.89 (95% CI 0.50–1.56) and 0.48 (95% CI 0.28–0.84), respectively.

**Conclusions:**

Decreased serum 25‐(OH)D level is significantly correlated with MetS in middle‐aged men.

## Graphical abstract

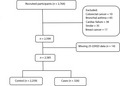

## Introduction

Metabolic syndrome (MetS) is a configuration of vascular risk factors, correlated with chronic conditions, such as type 2 diabetes mellitus and cardiovascular disease^1^. Recently, the prevalence of adults with MetS has increased globally by 25%^2^, and it is well established that the availability of MetS contributes to a greater risk of developing cardiovascular disease^3^, ^4^, suggesting that MetS is strongly linked with many non‐communicable diseases and it is continuously increasing the prevalence of such diseases. Thus, the prevention of this condition is crucial for public health.

Epidemiological studies show that serum concentrations of vitamin D are inversely associated with MetS^4^, ^5^. Vitamin D is known to help intestinal absorption of calcium and phosphorus^6^. A recent report showed that vitamin D deficiency reduces the intracellular calcium levels and thereby depletes insulin secretion level by β‐cells, which further impairs glucose tolerance^7^. Also, another study highlighted that vitamin D plays an essential role in glucose metabolism and insulin response by stimulating insulin receptors^8^. Increasing evidence suggests that vitamin D deficiency could be a risk factor for MetS^9^, ^10^. Similarly, a number of studies stated that higher vitamin D levels might have a protective effect against the development of MetS^11^, ^12^, ^13^, ^14^. For example, vitamin D supplementation has shown a significant drop in abdominal visceral adipose tissue in obese and overweight adults^15^. In addition, a 3‐month double‐blind randomized clinical trial of supplementations with vitamin D has shown a positive outcome in reducing body fat mass in women regardless of their body mass index (BMI) levels^16^. Recent clinical trials documented that vitamin D supplementation might improve ovulatory dysfunction and thereby revives fertility in women with polycystic ovarian syndrome^17^, ^18^. Similarly, some clinical and interventional studies explored the hypothesis that low serum vitamin D level is associated with the prevalence of MetS in postmenopausal women^19^, ^20^, ^21^, ^22^. Overall, these results support that there is an inverse correlation between vitamin D levels and the prevalence of MetS. Conversely, other studies failed to replicate this relationship among these specific groups of people^23^, ^24^. Thus, the reports on the inverse relationship between serum 25‐hydroxyvitamin D (25‐[OH]D) and MetS remained inconsistent. Furthermore, sex‐based evidence on the association of serum 25‐(OH)D levels with MetS in the middle‐aged and the older population is sparse.

In the present study, we investigated the association between serum 25‐(OH)D levels and the prevalence of MetS in the middle‐aged and elderly population of northeast China. Our results showed that there was a negative and persistent correlation between serum 25‐(OH)D concentrations and the prevalence of MetS in 2,585 participants, even after adjusting for the conventional risk factors of MetS.

## Methods

### Participants and methods

This was a comparative cross‐sectional study carried out from January 2017 to June 2018 in Dalian Port Hospital, Dalian, Liaoning, China. A total of 2,764 participants (1,511 women and 1,074 men) aged >50 years were enrolled from the Prospective Cohort Study of the Relative Factors of Heart Failure Among Elderly People data. The Prospective Cohort Study of the Relative Factors of Heart Failure Among Elderly People is a newly established prospective cohort study, currently registered in Dalian, China (ChiCTR‐1900021163), to investigate the risk factors for heart failure among the elderly population. The study conforms to the guidelines on biomedical research involving human participants (Declaration of Helsinki), and was approved by the ethics committee of the First Affiliated Hospital of Dalian Medical University (No. LCKY2016‐31). All participants provided written informed consent.

**Figure 1:**
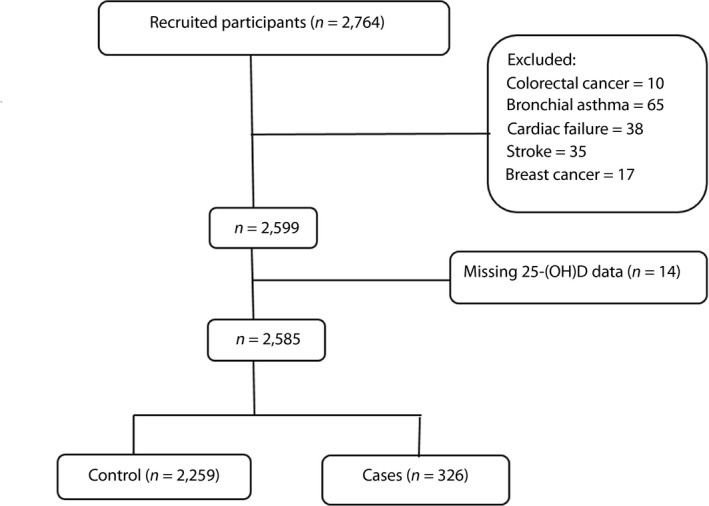
The flow chart of the recruitment and classification of the participants. 25‐(OH)D, 25‐hydroxyvitamin D.

We chose the definition of MetS based on the joint interim statement of the International Diabetes Federation and the American Heart Association based on the availability of any three of the following features: (i) waist circumference for Asians ≥80 cm in women and ≥90 cm in men; (ii) high‐density lipoprotein cholesterol (HDL) <50 mg/dL in women, <40 mg/dL in men; (iii) systolic blood pressure ≥130 mmHg or diastolic blood pressure ≥85 mmHg, (iv) fasting plasma glucose ≥100 mg/dL; and (v) triglycerides (TG) level ≥150 mg/dL^25^. All individuals aged >50 years with complete 25‐(OH)D data were eligible for the present study. The pre‐decided exclusion criteria were: chronic diseases that might potentially cause or affect the metabolism of vitamin D, cardiovascular risk due to existing or pre‐existing coronary heart disease, use of lipid‐lowering medications, chronic liver or renal disease, autoimmune disease, thyroid dysfunction, cancer, alcohol consumption and pharmacological use of vitamin D therapy. After excluding individuals who were not qualified for this study or had data errors, 2,585 participants were included in the analysis (Figure 1).

### Data collection

Data were collected at Dalian Port Hospital from January 2017 to June 2018. Physical examination was carried out for all participants, and their clinical results were carefully recorded and analyzed. A self‐administered questionnaire was also distributed to the participants to gather data on lifestyle, medical history, sociodemographic details and clinical characteristics. We determined BMI by dividing the weight in kilograms by the square of height in meters. The fasting (>8 h) plasma glucose level was assayed from the blood samples. Diabetes mellitus was diagnosed as a self‐reported history of diabetes, current use of antidiabetes agents, fasting plasma glucose levels of ≥7.0 mmol/L and/or hemoglobin A1c levels >7%. Two blood pressure readings were taken, using a blood pressure apparatus at 5‐min intervals on the right arm after a rest of 5 min with participants in the sitting position and the average of the two measurements was reported. Hypertension was diagnosed as systolic blood pressure >140 mmHg, diastolic blood pressure >90 mmHg and/or self‐reported use of antihypertensive drugs. Smoking was grouped into three classes: never smoker (never smoked in their lifetime), former smoker (ever smoking) and current smoker (smoking during the past year). The serum 25‐(OH)D concentration was determined by electrochemiluminescence immunoassay (ECLIA) (Roche Cobas e601, Indianapolis, IN). The levels of total cholesterol (TC), HDL, TG and serum uric acid were evaluated by standard enzymatic methods.

### Statistical analysis

Participants were categorized into quartiles based on the values of 25‐(OH)D (1st quartile ≤9.84 ng/mL, 2nd quartile 9.85–13.99 ng/mL, 3rd quartile 14.00–19.49 ng/mL and 4th quartile ≥19.5 ng/mL for the entire population; 1st quartile ≤12.08 ng/mL, 2nd quartile 12.08–16.65 ng/mL, 3rd quartile 16.66–22.90 ng/mL and 4th quartile ≥22.91 ng/mL for men; and 1st quartile ≤8.60 ng/mL, 2nd quartile 8.61–12.34 ng/mL, 3rd quartile 12.35–16.91 ng/mL and 4th quartile ≥16.92 ng/mL for women).

Data are presented as the mean ± standard deviation for all continuous variables, whereas counts and percentiles were used for categorical variables. The Mann–Whitney test was used to compare the differences in serum 25‐(OH) D concentrations between participants with and without MetS. The quartiles were calculated for the differences using the χ^2^‐test and analysis of variance (anova) for categorical and continuous variables, respectively. Preliminary analysis for serum 25‐(OH)D levels was carried out to ensure no violation of assumptions of normality, homoscedasticity, independence and linearity. Multicollinearity was assessed for the explanatory variables using tolerances and the variance inflation factors. The variance inflation factor of TC was >10, indicating that there were high correlations among the independent (predictor) variables. However, neither assumptions were violated nor multicollinearity was detected after the exclusion of TC from the model (Table S1). As such, the multiple linear regression analysis was adjusted for age, sex, cigarette status, alcohol consumption, physical activity, TG, HDL, LDL, BMI, creatinine and diabetes. Odds ratios (ORs) and 95% confidence intervals (CIs) were carried out for each quartile of serum level of 25‐(OH)D compared with the highest quartile value by using a logistic regression analysis model. The multivariable model was adjusted for sex, age, TC, LDL, creatinine, cigarette status, alcohol consumption and physical activity, whereas model 1 was unadjusted.

## Results

### Baseline characteristics

Demographic and basic clinical characteristics of participants are shown according to the quartiles of serum 25‐(OH)D levels in Table 1. The mean age, diastolic blood pressure and TG levels were markedly higher in the highest quartiles than in the first quartile. Additionally, participants with higher quartiles of serum 25‐(OH)D were older, less likely to engage in physical activity and more likely to have diabetes. However, the quartiles of serum 25‐(OH)D levels were similar with respect to body mass index, TC, LDL, HDL, FPG and systolic blood pressure (Table 1). No statistically significant difference was observed in antihypertensive drug use across the quartiles of serum 25‐(OH)D levels.

**Table 1:** Baseline characteristics of participants

Characteristic	Quartiles of 25‐(OH)D				*P*
	Quartile 1	Quartile 2	Quartile 3	Quartile 4	
Participants (*n*)	646	648	641	650	
Age, years (mean ± SD)	61.8 ± 5.81	61.7 ± 5.65	61.8 ± 5.75	62.6 ± 5.83	0.026
Male, *n* (%)	143 (22.1%)	238 (36.7%)	303 (47.3%)	390 (60.0%)	<0.001
BMI, kg/m^2^ (mean ± SD)	24.6 ± 3.25	24.8 ± 3.30	24.7 ± 3.15	24.9 ± 3.03	0.40
Current smoker (%)	74 (11.5%)	117 (18.1%)	129 (20.1%)	163 (25.1%)	<0.001
Alcohol drinkers, *n* (%)	79 (12.2%)	129 (19.9%)	166 (25.9%)	207 (31.8%)	<0.001
SBP, mmHg (mean ± SD)	145.7 ± 22.56	143.8 ± 21.77	143.7 ± 21.57	145.2 ± 21.77	0.240
DBP, mmHg (mean ± SD)	82.5 ± 12.37	83.5 ± 12.08	84.0 ± 12.57	85.6 ± 12.44	<0.001
Diabetes, *n* (%)	130 (20.1%)	136 (21.0%)	150 (23.4%)	152 (23.4%)	0.090
Plasma fasting glucose (mg/dL)	6.2 ± 1.94	6.2 ± 1.96	6.2 ± 1.79	6.2 ± 1.75	0.900
Physical activity, *n* (%)	268 (41.5%)	267 (41.2%)	270 (42.1%)	328 (50.5%)	<0.001
TC, mmol/L (mean ± SD)	5.5 ± 1.03	5.4 ± 0.99	5.5 ± 1.01	5.4 ± 0.98	0.080
TG, mmol/L (mean ± SD)	1.7 ± 1.65	1.5 ± 0.98	1.5 ± 0.99	1.4 ± 0.76	<0.001
HDL‐c, mmol/L (mean ± SD)	1.4 ± 0.34	1.5 ± 0.38	1.5 ± 0.37	1.5 ± 0.38	0.500
LDL‐c, mmol/L (mean ± SD)	3.5 ± 0.81	3.4 ± 0.83	3.4 ± 0.83	3.4 ± 0.83	0.200
Creatinine, μmol/L (mean ± SD)	3.4 ± 0.83	62.0 ± 12.67	64.0 ± 12.21	67.4 ± 13.87	<0.001
Hypertension	376 (58.2%)	350 (54.0%)	355 (55.4%)	391 (60.2%)	0.400
Anti‐hypertensive use, *n* (%)	99 (15.3%)	84 (13.0%)	79 (12.3%)	95 (14.6%)	0.650
β‐Blockers, *n* (%)	18 (2.8%)	12 (1.9%)	13 (2.0%)	23 (3.5%)	0.380
ACEI, *n* (%)	4 (0.6%)	3 (0.5%)	6 (0.9%)	3 (0.5%)	1.000
ARB, *n* (%)	22 (3.4%)	21 (3.2%)	15 (2.3%)	17 (2.6%)	0.270
Calcium antagonists, *n* (%)	43 (6.7%)	41 (6.3%)	41 (6.4%)	39 (6.0%)	0.660
Diuretics, *n* (%)	2 (0.3%)	0 (0.0%)	3 (0.5%)	0 (0.0%)	0.550
Other antihypertensive use, *n* (%)	22 (3.4%)	15 (2.3%)	15 (2.3%)	26 (4.0%)	0.540

Total *n* = 2,585. 25‐(OH)D, 25‐hydroxyvitamin D; ACEI, angiotensin‐converting enzyme inhibitors; ARB, angiotensin receptor blockers; BMI, body mass index; DBP, diastolic blood pressure; HDL‐c, high‐density lipoprotein; LDL‐c, low‐density lipoprotein; SBP, systolic blood pressure; TC, total cholesterol; TG, triglycerides.

### Comparison of serum 25‐(OH)D levels between the Mets and control groups

Serum 25‐(OH)D values were expressed as the median (25% quartiles to 75% quartiles). The MetS participants had lower serum 25‐(OH)D levels (*P *=* *0.007) compared with the control group in men (Table 2). However, the serum 25‐(OH)D levels were similar between the Mets and control groups in the entire population and women category.

**Table 2:** Comparison of serum 25‐hydroxyvitamin D levels between metabolic syndrome and control groups

Target group	Total	Cases	Control	*P*
General population	2,585	*n* = 326	*n* = 2,259	0.124
		13.16 (9.13–18.98)	14.12 (9.96–19.62)	
Men	1,074	*n* = 150	*n* = 924	0.007
		15.82 (11.32–19.74)	16.83 (12.17–23.75)	
Women	1,511	*n* = 176	*n* = 1,335	0.393
		11.53 (8.26–17.58)	12.45 (8.65–16.90)	

### Association between serum 25‐(OH)D concentrations and lipid profile, and other risk factors of MetS

Table S1 shows the associations between serum 25‐(OH)D levels and lipid profile, and other risk factors of MetS. Serum 25‐(OH)D levels were positively associated with physical activity, HDL, LDL, creatinine and BMI. However, there was an inverse association between serum 25‐(OH)D and sex (β = −0.266, 95% CI −5.278 to −3.118; *P* < 0.001), and TG (β = −0.058, 95% CI −0.669 to −0.120; *P *=* *0.005). The association between serum 25‐(OH)D and sex showed that there was a need for sex‐based analysis when examining the predictive power of serum 25‐(OH)D levels and the presence of MetS.

### Relationship between serum 25‐(OH)D levels and the risk of MetS

When serum 25‐(OH)D concentrations were used as continuous data, binary logistic regression analysis showed a negative correlation between serum levels of 25‐(OH)D and the prevalence of MetS in the category of the entire population and men. The correlation between serum level of 25‐(OH)D and MetS was persisted even after potential confounders were added to the model (model 3 in Table 3).

**Table 3:** Relationship between serum 25‐hydroxyvitamin D levels and the risk of metabolic syndrome

	Entire population			Women			Men		
	OR	95% CI	*P*	OR	95% CI	*P*	OR	95% CI	*P*
Model 1	0.99	0.97–1.00	0.046	1.00	0.97–1.02	0.83	0.97	0.94–0.99	<0.01
Model 2	0.98	0.96–1.00	0.01	1.00	0.98–1.02	0.88	0.96	0.94–0.99	<0.01
Model 3	0.98	0.96–0.99	0.01	1.00	0.97–1.02	0.84	0.96	0.94–0.98	<0.01

Model 1: unadjusted. Model 2: adjusted for age, sex, cigarette smoking status, alcohol consumption and physical activity. Model 3: adjusted for the aforementioned plus total serum cholesterol, low‐density lipoprotein cholesterol and creatinine.CI, confidence interval; OR, odds ratio.

### Risk of MetS in the entire population according to the quartiles of serum 25‐(OH)D levels

The correlation between quartiles of serum 25‐(OH)D level and MetS is shown in Table 4. Compared with participants in the first quartile, the positive and persistent correlations were observed between the higher quartiles of serum 25‐(OH)D levels and MetS in the entire population. This correlation persisted after adjusted for other potential confounders including age, cigarette smoking status, alcohol consumption, physical activity, TC, LDL and creatinine.

**Table 4:** Risk of metabolic syndrome in the entire population according to the quartiles of serum 25‐hydroxyvitamin D levels

	Quartiles of 25‐(OH)D in the entire population			
	Quartile 1	Quartile 2	Quartile 3	Quartile 4
Cut‐off points	≤9.84	9.85–13.99	14.00–19.49	≥19.5
Model 1	1.000 (ref.)	0.78 (0.57–1.08)	0.77 (0.58–1.09)	0.74 (0.53–1.02)
Model 2	1.000 (ref.)	0.76 (0.55–1.05)	0.73 (0.53–1.02)	0.65 (0.47–0.92)*
Model 3	1.000 (ref.)	0.76 (0.55–1.06)	0.74 (0.53–1.03)	0.67 (0.45–0.90)*

Model 1: unadjusted. Model 2: adjusted for l physical activity. Model 3: adjusted for the aforementioned plus total serum cholesterol, low‐density lipoprotein cholesterol and creatinine. *P <* *0.05. 25‐(OH)D, 25‐hydroxyvitamin D.

### Risk of MetS in men and women according to the quartiles of serum 25‐(OH)D levels

The correlation between quartiles of serum 25‐(OH)D levels and MetS is shown in Table 5. Compared with participants in the first quartile, the positive and persistent correlations between the higher quartiles of serum 25‐(OH)D levels and MetS among men were observed. Importantly, this relationship was maintained even after multivariable adjustment for potential confounders.

**Table 5:** Risk of metabolic syndrome in men and women according to the quartiles of serum 25‐hydroxyvitamin D levels

	Quartiles of 25‐(OH)D in men			
	Quartile 1	Quartile 2	Quartile 3	Quartile 4
Cut‐off points	≤12.08	12.08–16.65	16.66–22.90	≥22.91
Model 1	1.000 (ref.)	0.93 (0.55–1.58)	0.87 (0.50–1.52)	0.51 (0.30–0.88)*
Model 2	1.000 (ref.)	0.90 (0.53–1.53)	0.86 (0.49–1.51)	0.50 (0.29–0.87)*
Model 3	1.000 (ref.)	0.93 (0.54–1.59)	0.89 (0.50–1.56)	0.48 (0.28–0.84)*

	Quartiles of 25‐(OH)D in women			
Cut‐off points	≤8.60	8.61–12.34	12.35–16.91	≥16.92
Model 1	1.000 (ref.)	0.68 (0.45–1.02)	0.55 (0.35–0.87)*	0.98 (0.63–1.51)
Model 2	1.000 (ref.)	0.68 (0.45–1.02)	0.56 (0.35–0.89)*	0.98 (0.63–1.53)
Model 3	1.000 (ref.)	1.04 (0.66–1.63)	0.71 (0.43–1.16)	0.58 (0.34–0.99)*

Model 1: unadjusted. Model 2: adjusted for age, cigarette smoking status, alcohol consumption and physical activity. Model 3: adjusted for the above plus total serum cholesterol, low‐density lipoprotein cholesterol and creatinine. *P <* *0.05. 25‐(OH)D, 25‐hydroxyvitamin D.

### Relationship between serum 25‐(OH)D concentrations and Mets based on age category

The participants were categorized into two groups by their age: ≤65 and ≥65 years. The correlation between serum 25‐(OH)D levels and MetS persisted in men who were aged <65 years, but not in participants aged >65 years, implying that serum 25‐(OH)D could be a potential marker for the risk prediction of MetS in the middle‐aged men. In an unadjusted model, a negative correlation was observed between serum 25‐(OH)D levels and MetS among older participants (aged ≥65 years) in the category of the entire population and men. Nevertheless, this correlation was not sustained after adjusted for other potential confounders (Table 6).

**Table 6:** The relationship between serum 25‐hydroxyvitamin D levels and metabolic syndrome based on age categories

	Entire populations		Men		Women	
	<65 years	>65 years	<65 years	>65 years	<65 years	>65 years
	OR (95% CI)	OR (95% CI)	OR (95% CI)	OR (95% CI)	OR (95% CI)	OR (95% CI)
Unadjusted	0.99 (0.97–1.01)	0.97 (0.94–1.00)*	0.97 (0.94–1.00)*	0.96 (0.92–1.00)*	1.00 (0.97–1.02)	0.98 (0.93–1.05)
Adjusted	0.98 (0.95–1.00)	0.96 (0.91–1.01)	0.96 (0.92–1.00)*	0.97 (0.90–1.04)	0.99 (0.96–1.03)	0.96 (0.88–1.06)

*P <* *0.05. 25‐(OH)D, 25‐hydroxyvitamin D; CI, confidence interval; OR, odds ratio.

## Discussion

In the present cross‐sectional study of 2,585 individuals, we found that there was a negative correlation between circulating 25‐(OH)D and MetS in the middle‐aged men in Dalian Port Hospital, Dalian, Liaoning, China. The correlation between serum 25‐(OH)D concentrations and MetS remained after adjusting for the conventional risk factors of MetS.

Previous studies showed that the correlations between the serum vitamin D or 25‐(OH)D concentrations and MetS are controversial. For example, there is an inverse correlation between the lowered serum 25‐(OH)D concentrations and MetS in postmenopausal women^20^. Vitamin D intake is inversely related to a risk of MetS in African American and white men and women^26^. On the contrary, serum 25‐(OH)D concentrations were negatively associated with the risk of elevated blood pressure and MetS in middle‐aged and older Korean adults^12^. Furthermore, higher serum 25(OH)D concentrations are associated with a lower prevalence of MetS in the elderly^27^. In the present study, we found that the median concentration of serum 25‐(OH)D was markedly lower in the MetS group than in the control group in men (Table 2). However, the median concentration of serum 25‐(OH)D level was similar between the MetS group and control group in women and the entire population (Table 2). In addition, there was a significant correlation between serum 25‐(OH)D levels and MetS in men, but not in women (Table 3). In this sense, the present results were in agreement with the previous reports^23^, ^28^, ^29^, ^30^. Interestingly, we found that the median value of serum 25‐(OH)D in the MetS group was decreased in women compared with the control group, but there was no significant difference between two groups (Table 2). According to the previous evidence, it is well established that women at the postmenopausal stage are at higher risk of MetS because of hormonal changes^31^. Estrogen promotes the enzyme activity that is responsible for activating vitamin D^32^. The reduction in the levels of estrogen during the transition of the menopausal stage could result in clinical manifestations of vitamin D deficiency^32^. According to the published reports, there is a significant increase in visceral fat mass in postmenopausal women^33^. This is mainly because of the impact of estrogen on lipogenesis and lipolysis in visceral adipocytes in women. However, depending on our result, the body mass index of most women has fallen within the normal range (Table 1), and that could be the reason for the absence of a correlation between serum 25‐(OH)D levels and MetS in women. Furthermore, the inverse correlation between serum 25‐(OH)D concentrations and MetS in men and not in women could be elucidated by the difference in age, visceral adiposity, lean muscle, free fatty acid‐induced peripheral insulin resistance, hormonal regulation of bodyweight and lifestyle (physical exercise and vitamin supplementation), and an influence of the menopausal change^34^. Consequently, additional studies are required to cautiously control the effect of body mass index to avoid bias related to sex differences.

Although the role of vitamin D deficiency in the pathogenesis of MetS is not well elucidated, vitamin D supplementation was found to be effective in the components of MetS. Vitamin D supplementation contributes to the hormonal treatment of serum lipid level, decreasing the risk of MetS^35^. Furthermore, type 2 diabetes individuals had a significantly lowered serum 25‐(OH)D concentrations compared with controls due to the existence of vitamin D receptors on pancreatic β*‐*cells and other insulin‐sensitive tissue^36^. Also, vitamin D supplementation for patients with type 2 diabetes improved fasting plasma glucose and the insulin sensitivity index^37^. Similarly, an experimental study showed that vitamin D administration inhibited secretion of parathyroid hormone^38^, and lower levels of which have been associated with improved insulin sensitivity in humans^39^. Health policy‐makers should take note that lower circulating levels of serum 25‐(OH)D do not only inform the status of vitamin D deficiency, but might also predict the risk of MetS in men of middle‐aged categories. Therefore, the present study merits a prospective study to examine circulating levels of serum 25‐(OH)D as a potential marker for MetS risk stratification. In conclusion, the present study suggests an inverse correlation between serum 25‐(OH)D levels and MetS in men.

In the present study, there were several limitations, including: (i) long‐term follow up is required to understand the causal relationship between vitamin D and MetS; (ii) the lack of data on serum calcium, phosphate and parathyroid hormone, and the relationships between these parameters and serum 25‐(OH)D^39^, ^40^; and (iii) although ECLIA is a rapid and precise assay for serum 25‐(OH)D measurement over a wide range on automated immunoassay platforms^41^, the serum 25‐(OH)D levels are required to be confirmed using the liquid chromatography–mass spectromoetry (LC‐MS) method, and to verify the associations between serum 25‐(OH)D levels and MetS in the future. It is not uncommon to observe that several studies prefer to assess serum 25‐(OH)D using the LC‐MS method over ECLIA^42^, ^43^. The use of both the LC‐MS and ECLIA methods would have strengthened the validity of our associations; for example, differences in these methods (if any) could be of importance when examining the concentrations of 25‐(OH)D. However, the present study did not consider the LC‐MS approach, because it was virtually unsuitable in our laboratory where this technique was unaffordable due to its high cost and the nature of the large sample size. In addition, ECLIA is recommended as a viable alternative for routine 25‐(OH)D assessment, following that its results were found to be accurate and ultimately correlated with the LC‐MS method^44^. Also, ECLIA is a rapid and precise assay for 25‐(OH)D serum measurement over a wide range on automated immunoassay platforms^41^, suggesting that ECLIA technique is an effective approach to determine the serum 25‐(OH)D levels.

## Disclosure

The authors declare no conflict of interest.

## Supporting information


**Table S1**| Associations between serum 25‐hydroxyvitamin D levels and lipid profile, and other risk factors of metabolic syndrome.
