# Supplementary material for: Association between serum 25‐hydroxyvitamin D concentrations and metabolic syndrome in the middle‐aged and elderly Chinese population in Dalian, northeast China: A cross‐sectional study
Source: J Diabetes Investig. 2019 Jun 21;11(1):184–91. doi: 10.1111/jdi.13086 (PMC6944851; doi:10.1111/jdi.13086)
Supplement: Supplementary file 1 — Table S1| Associations between serum 25‐hydroxyvitamin D levels and lipid profile, and other risk factors of metabolic syndrome. [file JDI-11-184-s001.doc]

**Table S1: The associations between serum 25-(OH)D levels and lipid profile, and other risk factors of MetS.**

|  | Unstandardized Coefficients | | Standardized Coefficients |  |  | 95.0% Confidence Interval for B | | Collinearity Statistics | |
| --- | --- | --- | --- | --- | --- | --- | --- | --- | --- |
|  | β | SE | β | t | Sig. | Lower Bound | Upper Bound | Tolerance | VIF |
| (Constant) | 13.502 | 3.076 |  | 4.389 | .000 | 7.469 | 19.535 |  |  |
| gender | -4.198 | .551 | -.266 | -7.621 | .000 | -5.278 | -3.118 | .288 | 3.474 |
| Age (years) | -.017 | .027 | -.012 | -.627 | .531 | -.069 | .035 | .903 | 1.107 |
| Alcohol drinking | .184 | .237 | .020 | .775 | .438 | -.281 | .649 | .539 | 1.855 |
| Cigarette status | -.222 | .276 | -.023 | -.807 | .420 | -.763 | .318 | .448 | 2.233 |
| Physical activity | .899 | .226 | .076 | 3.973 | .000 | .455 | 1.342 | .961 | 1.041 |
| HDL (mmol/L) | 1.463 | .465 | .069 | 3.149 | .002 | .552 | 2.374 | .725 | 1.380 |
| LDL (mmol/L) | .405 | .183 | .043 | 2.207 | .027 | .045 | .764 | .927 | 1.079 |
| TG (mmol/L) | -.394 | .140 | -.058 | -2.816 | .005 | -.669 | -.120 | .821 | 1.218 |
| Creatinine (μmol/L) | .034 | .014 | .059 | 2.347 | .019 | .006 | .062 | .547 | 1.828 |
| BMI (kg/m^2^) | .102 | .049 | .042 | 2.064 | .039 | .005 | .198 | .864 | 1.157 |
| DM | -.009 | .367 | .000 | -.025 | .980 | -.728 | .710 | .920 | 1.087 |

HDL-c, High-density lipoprotein; LDL-c, Low-density lipoprotein; TG, Triglycerides;BMI, body mass index;DM, diabetes mellitus.
